# What have we learned from a case of convalescent plasma treatment in a two-time kidney transplant recipient COVID-19 patient? A case report from the perspective of viral load evolution and immune response

**DOI:** 10.3389/fneph.2023.1132763

**Published:** 2023-06-23

**Authors:** Fabian Aldunate, Alvaro Fajardo, Natalia Ibañez, Florencia Rammauro, Hellen Daghero, Rodrigo Arce, Diego Ferla, Marianoel Pereira-Gomez, Cecilia Salazar, Gregorio Iraola, Otto Pritsch, Javier Hurtado, Jordan Tenzi, Mariela Bollati-Fogolín, Sergio Bianchi, Nicolas Nin, Gonzalo Moratorio, Pilar Moreno

**Affiliations:** ^1^ Laboratorio de Virología Molecular, Centro de Investigaciones Nucleares, Facultad de Ciencias, Universidad de la República, Montevideo, Uruguay; ^2^ Laboratorio de Evolución Experimental de Virus, Institut Pasteur Montevideo, Montevideo, Uruguay; ^3^ Laboratorio de Inmunovirología, Institut Pasteur de Montevideo, Montevideo, Uruguay; ^4^ Departamento de Inmunobiología, Facultad de Medicina, Universidad de la República, Montevideo, Uruguay; ^5^ Cell Biology Unit, Institut Pasteur de Montevideo, Montevideo, Uruguay; ^6^ Laboratorio de Genómica Microbiana, Institut Pasteur de Montevideo, Montevideo, Uruguay; ^7^ Host-Microbiota Interactions Laboratory, Wellcome Sanger Institute, Hinxton, United Kingdom; ^8^ Unidad de Cuidados Intensivos, Hospital Español “Juan José Crottoggini”, Administración de Servicios de Salud del Estado, Montevideo, Uruguay; ^9^ Laboratorio de Biomarcadores Moleculares, Departamento de Fisiopatología, Hospital de Clínicas, Universidad de la República, Montevideo, Uruguay; ^10^ Laboratorio de Genómica Funcional, Institut Pasteur de Montevideo, Montevideo, Uruguay

**Keywords:** COVID-19, kidney transplant, convalescent plasma therapy, SARS-CoV-2, molecular diagnosis

## Abstract

Coronavirus disease 2019 (COVID-19), an infectious disease caused by the severe acute respiratory syndrome coronavirus 2 (SARS-CoV-2) virus, can have a wide range of clinical manifestations, ranging from asymptomatic disease to potentially life-threatening complications. Convalescent plasma therapy has been proposed as an effective alternative for the treatment of severe cases. The aim of this study was to follow a two-time renal transplant patient with severe COVID-19 treated with convalescent plasma over time from an immunologic and virologic perspective. A 42-year-old female patient, who was a two-time kidney transplant recipient, was hospitalized with COVID-19. Due to worsening respiratory symptoms, she was admitted to the intensive care unit, where she received two doses of convalescent plasma. We analyzed the dynamics of viral load in nasopharyngeal swab, saliva, and tracheal aspirate samples, before and after convalescent plasma transfusion. The levels of pro-inflammatory cytokines and antibody titers were also measured in serum samples. A significant decrease in viral load was observed after treatment in the saliva and nasopharyngeal swab samples, and a slight decrease was observed in tracheal aspirate samples. In addition, we found evidence of an increase in antibody titers after transfusion, accompanied by a decrease in the levels of several cytokines responsible for cytokine storm.

## Introduction

Coronavirus disease 2019 (COVID-19), caused by severe acute respiratory syndrome coronavirus-2 (SARS-CoV-2), has become a major global health concern. To date, this virus has infected more than 500 million people worldwide, resulting in more than 6.3 million reported deaths (1).

Clinical manifestations of COVID-19 vary widely among patients, ranging from mild disease to severe respiratory failure that can lead to death. COVID-19 induces an exaggerated and misdirected activation of the immune system leading to inflammation characterized mainly by high levels of pro-inflammatory cytokines, known as a cytokine storm (CS) (2). Convalescent plasma (CP) is a passive immunization strategy used in the treatment of various infectious diseases. CP transfusion provides neutralizing antibodies and other immune components with the goal of reducing the risk of disease and attenuating severe inflammatory responses. It has been reported that early administration of high-titer CP against SARS-CoV-2 to mildly ill infected older adults reduces the progression of COVID-19 (3). However, little is known about the clinical course and molecular response in immunosuppressed patients, such as solid organ transplant (SOT) recipients, infected with SARS-CoV-2, or about the optimal management of such patients.

In this article, we report a complete case of COVID-19 infection in a two-time kidney transplant recipient (KTR) treated with CP, with complete information on the clinical course, viral load dynamics, and immune response evolution. The aim of this report is to better characterize, from a virological and immunological perspective, the response of a two-time kidney transplant patient treated with CP.

## Case description

A 42-year-old woman with end-stage kidney disease due to reflux nephropathy received her second deceased-donor kidney transplant in 2018. At that time, the patient exhibited a negative complement-dependent cytotoxicity crossmatch reaction, but human leukocyte antigen (HLA) antibody testing revealed the presence of donor-specific antibodies of HLA classes I and II. Therefore, she underwent desensitization procedures, including plasmapheresis, post-transplant rituximab and immunoglobulin, and anti-thymocyte globulin induction. Her post-transplant course was uncomplicated, without kidney allograft rejection or infectious disease, and she had a baseline serum creatinine level of 0.9 mg/dL. She continued with maintenance immunosuppression therapy with tacrolimus (2.5 mg/12 h), mycophenolate sodium (360 mg/12 h), and oral corticosteroids (prednisone 5 mg/day).

On 15 October 2020 (day 1 since onset of symptoms), the patient presented with cough, expectoration, dysgeusia, and anosmia. Oxygen saturation and chest radiography were performed, prior to prescribing azithromycin, with normal results. Azithromycin treatment was started empirically (500 mg/day), and the patient was sent home with isolation instructions. The patient was not vaccinated against SARS-CoV-2 because the vaccines were not available at that time.

On 23 October, the patient tested positive for SARS-CoV-2 by reverse transcription polymerase chain reaction (RT-PCR) analysis of a nasopharyngeal swab (NS) sample collected on 21 October. On that day, the patient was admitted to the hospital for close monitoring and further testing given the appearance of dyspnea on minimal exertion, fever, and her previous medical history. Physical examination on the day of admission revealed a body temperature of 39.0°C, blood pressure of 140/80 mmHg, heart rate of 90 bpm, blood oxygen saturation of 98% on room air without immunosuppression stigmas, and breath sounds on chest auscultation. High-resolution chest computed tomography and chest radiography were performed. The results of these tests showed multiple patchy ground-glass opacities in bilateral subpleural areas. Bibasilar atelectasis was also observed. At that time, C-reactive protein (CRP) (2.7 mg/dL) and D-dimer (0.43 ng/mL) levels were within normal limits. Blood counts were also normal: white blood cell count was 4,500/mm^3^, hemoglobin level was 13.1 g/dL, and platelet count was 176,000/mm^3^. The function of the allograft was also stable, with a creatinine level of 0.9 mg/dL. Oral administration of tacrolimus and mycophenolate mofetil was discontinued, and oral corticosteroids were replaced with 8 mg/day of dexamethasone. Empirical antibiotics were started with piperacillin/tazobactam.

**Figure 1:**
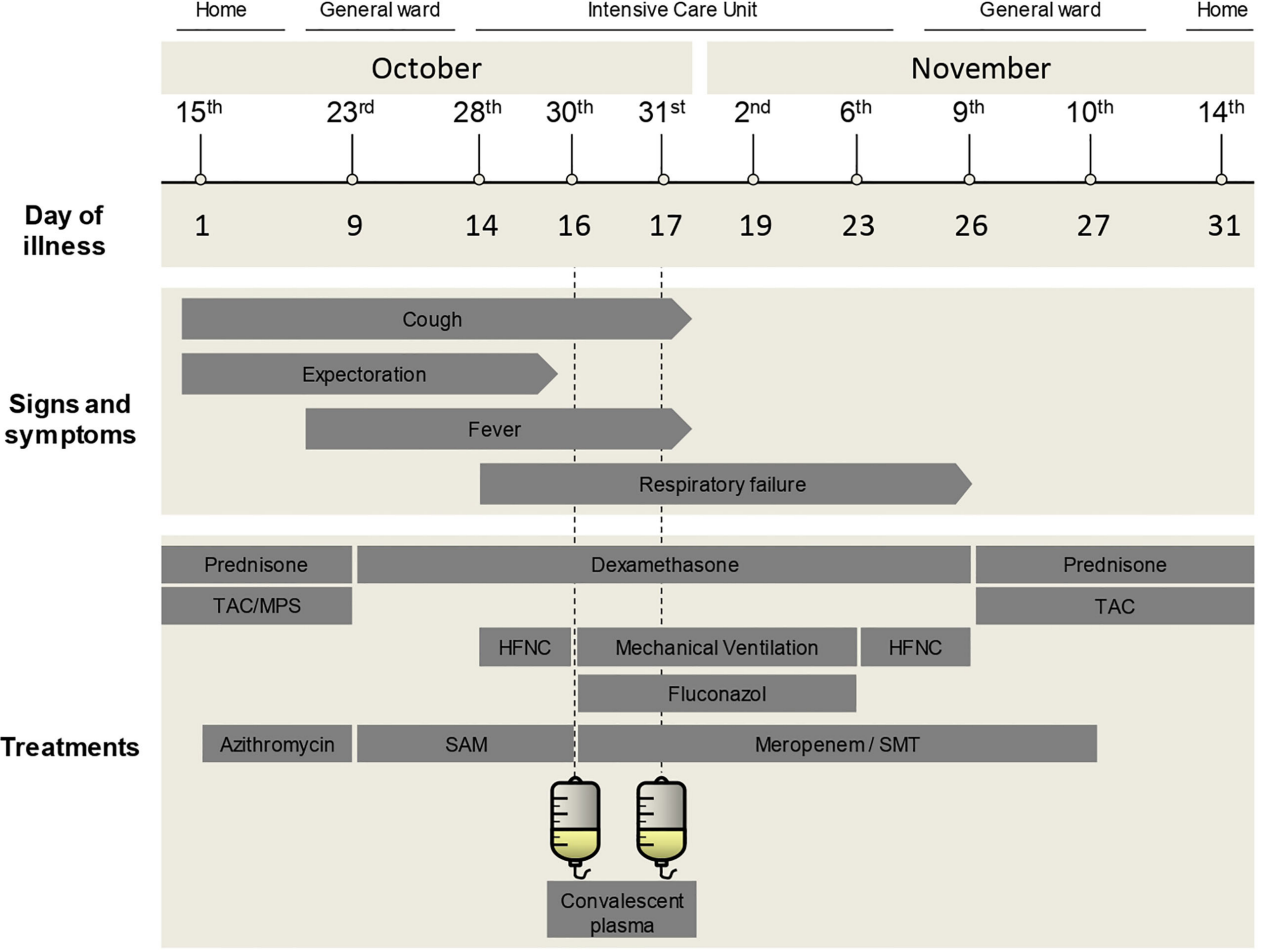
Medical history timeline. Timeline of the case showing main time points, day of illness, signs and symptoms, and treatments received during admission. TAC, tacrolimus; MPS, mycophenolate sodium; SAM, ampicillin/sulbactam; SMT, trimethoprim–sulfamethoxazole; HFNC, high-flow nasal cannula.

On 28 October, 13 days after the onset of symptoms, the patient presented with worsening respiratory symptoms and was admitted to the intensive care unit (ICU). She was found to be severely hypoxic, with a respiratory rate of 25 breaths per minute and a pulse oximeter reading of 90% on room air. She was treated with a high-flow nasal cannula without improvement in gas exchange. Arterial blood gas confirmed acute hypoxic respiratory failure and she was orotracheally intubated with ventilatory support. At this critical point, the patient received two doses of CP 24 h apart (30 and 31 October), as shown in **Figure 1**. The infused CP had a median titer of 1:3,200 of total SARS-CoV-2 antibodies (interquartile range 1:800 to 1:3,200). In addition, antibiotic treatment was empirically changed to meropenem (1 g/8 h), trimethoprim–sulfamethoxazole (1 amp/12 h), and fluconazole (200 mg/12 h), but all cultures were negative. Blood, urine, and tracheal aspirate cultures yielded no microbial growth. In addition, a serum galactomannan antigen test was negative; cytomegalovirus viral load was found to be undetectable; immunofluorescence testing for *Pneumocystis jirovecii* was negative; and a GeneXpert® Bacilo Koch test was negative, as well as tests for influenza and atypical viruses. The patient also underwent bronchoalveolar lavage, which was negative for bacterial cultures, and tested positive for *Candida albicans*.

After CP transfusion, the clinical evolution of the patient’s condition was favorable. On 6 November (day 23 since the onset of symptoms) the patient’s clinical respiratory status and oxygen saturation improved. She was extubated without any complications and treatment with fluconazole was suspended. Three days later, her general clinical condition improved and tacrolimus was restarted (1 mg/12 h). The patient was able to breathe unassisted with oxygen saturation above 97%. She was discharged on 14 November (31 days after the onset of symptoms). Currently, the patient is asymptomatic with no sequelae of long COVID and has completed the vaccination schedule, which for this type of immunocompromised patient is five doses of the mRNA vaccine. **Supplementary Table 1** shows paraclinical values before, during, and after the CP transfusion.

## SARS-CoV-2 viral load, specific antibody levels, and cytokine dynamics after convalescent plasma treatment

Nasopharyngeal swab (NS), saliva, and tracheal aspirate (TA) samples were collected from the patient before, during, and after treatment with CP. Serum samples were also collected at the same time points. Viral RNA was extracted using a QIAamp® Viral RNA Mini Kit (QIAGEN, Hilden, Germany) in accordance with the manufacturer’s protocol. Detection of SARS-CoV-2 was performed with the national “COVID-19 RT-PCR Real TM Fast-HEX/Cy5 Kit” in accordance with the manufacturer’s protocol (ATGen, Institut Pasteur de Montevideo, Universidad de la República, Montevideo, Uruguay).

**Figure 2:**
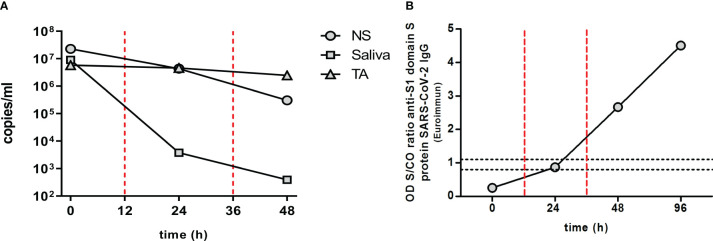
Viral load and antibody dynamics. **(A)** Viral load dynamics: SARS-CoV-2 viral load in nasopharyngeal swab (NS), saliva, and tracheal aspirate (TA) over time. **(B)** Anti-SARS-CoV2 antibody dynamics: specific anti-S1 domain of spike (S) protein SARS-CoV-2 IgG antibody detected in patient sera during convalescent plasma (CP) treatment. Results are shown in the form of an OD sample/cutoff value (S/CO) ratio. Samples with an S/CO ratio below 0.8 are considered negative, above 1.1 positive, and between 0.8 and 1.1 equivocal. The red vertical dashed lines indicate the timing of CP transfusions. COVID-19, coronavirus disease 2019; Ig, immunoglobulin; SARS-CoV-2, severe acute respiratory syndrome coronavirus 2.

A decrease in viral load was observed in the NS, saliva, and TA samples after CP transfusion (**Figure 2A**). This decrease was most pronounced in the case of the saliva samples, followed by NS samples. TA samples also showed a decrease in viral load after treatment, but to a lesser extent. The complete SARS-CoV-2 sequence was obtained from all samples collected (NS, saliva, and TA) using the MinION™ platform (Oxford Nanopore Technologies, Oxford, UK). It was assigned to the B.1.1.33 SARS-CoV-2 lineage (see **Supplementary Figure 1**).

Saliva and NS samples were negative at 72 h; TA sampling was not performed at this time point due to the clinical improvements in the patient’s condition.

**Figure 3:**
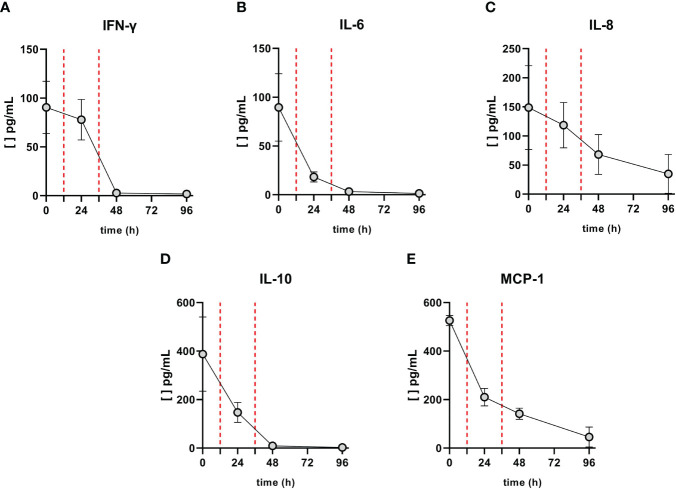
Levels of cytokines before and after convalescent plasma (CP) transfusions. The concentration of **(A)** IFN-γ, **(B)** IL-6, **(C)** IL-8, **(D)** IL-10, and **(E)** MCP-1 were measured by LEGENDplex™ Human Inflammation Panel 1 (BioLegend®, San Diego, CA, USA). Red vertical dashed lines indicate when the CP transfusions were performed. IFN, interferon; IL, interleukin; MCP-1, monocyte chemotactic protein 1.

Anti-RBD (Wuhan variant) IgG serum levels were quantified using the COVID-19 IgG ELISA kit (developed by the Universidad de la República, Institut Pasteur de Montevideo, and ATGen SRL) in accordance with the manufacturer’s instructions. An increase in specific antibodies was observed after CP treatment, which correlated with the decrease in viral load (**Figure 2B**). Prior to treatment, no anti-SARS-CoV-2 antibodies were detected in the patient’s serum (measurements below the detection limit). However, 12 h after the second CP administration, specific antibodies were detected, with up to a threefold change in the sample/cutoff (S/CO) ratio being measured. This increase in circulating anti-SARS-CoV-2 antibodies was further demonstrated at 84 h after the first dose (60 h after the second CP administration), reaching a 4.5-fold change in ratio. In addition, a decrease in informative pro-inflammatory cytokines involved in the CS was measured in the patient after CP treatment (**Figure 3**). Decreases in the levels of interferon gamma (IFN-γ), interleukin 8 (IL-8), interleukin 10 (IL-10), and chemoattractant protein-1 (MCP-1) were observed 12 h after the first CP transfusion (30 October), with a sharp decrease in interleukin 6 (IL-6). At 12 h after the second CP transfusion, IFN-γ, IL-6, and IL-10 became undetectable.

## Discussion and conclusions

An exaggerated immune response is a main cause of severe COVID-19 cases. CS is characterized by the overproduction of a number of pro-inflammatory cytokines and is strongly associated with poor prognosis (4). During the current COVID-19 pandemic, before vaccines and antiviral drugs were available, CP was widely used at various stages of the disease. The evidence for the use of CP in patients with severe COVID-19 infection is inconclusive, and some reports have questioned its efficacy due to the small sample sizes and lack of control groups in some studies (5, 6). A randomized trial showed no significant differences in clinical status or overall mortality between patients treated with CP and those who received placebo (7). However, it is important to highlight that these reports do not focus on immunocompromised patients, such as solid organ transplant recipients. On the other hand, there is increasing evidence that CP administration is safe and may lead to more favorable clinical outcomes and reduced mortality when used as early as possible in the early stages of the disease. However, large high-quality studies are needed to establish robust evidence for CP therapy (3, 8–11). It is also important to mention that CP treatment may cause potential adverse events in some patients, such as worsening hypoxemia and respiratory failure; transfusion-related complications have also been reported (12).

Solid organ transplant recipients and patients with underlying kidney disease represent a vulnerable population at high risk for adverse outcomes from COVID-19. These populations may have deficiencies in clearing the SARS-CoV-2 virus and develop more aggressive disease, yet they have been excluded or underrepresented in many clinical trials. There are randomized clinical trials suggesting that the administration of CP may be useful in patients with some types of immunodeficiency (13). This could be explained by the fact that immunocompromised patients may not have developed endogenous antibody responses. There is also evidence that kidney transplant recipients exhibit a poor serologic response to mRNA vaccination, especially those on triple immunosuppression (14). There are several reported cases of transplant recipients with COVID-19 who received CP transfusions and subsequently made a good recovery without adverse reactions (15). The goal of these therapies is to reduce the exaggerated inflammatory response observed in this type of patient. Most of these transplanted patients have had successful outcomes without disease progression (16–19). A cohort study showed no difference in the outcomes of kidney transplant recipients with mild and severe COVID-19 treated with CP compared with a control group. However, the study used a small sample size and a low proportion of high-titer CP, and included a very small proportion of patients who had received two kidney transplants (20).

The efficacy of CP therapy in the treatment of COVID-19 depends on several factors, including the level of neutralizing antibodies in the donor plasma (which can vary between individuals and with the timing of plasma collection), the timing of treatment, and the severity of the disease. The emergence of SARS-CoV-2 genomic variants may also affect the efficacy of CP treatment. Although it is not yet clear how effective CP therapy will be against newer variants, some studies suggest that CP therapy with plasma from individuals who have recovered from the original strain may be less effective against some of the new variants, such as the beta and gamma variants (21).

Several therapeutics for COVID-19 have been developed since the onset of the pandemic in early 2020. The development of monoclonal antibodies as a treatment and prophylaxis strategy for COVID-19 has provided an alternative to CP therapy, as these antibodies are effective against a range of SARS-CoV-2 variants. However, some, but not all, of the antibodies in clinical use may lose efficacy to some omicron sublineages (22).

Other drugs, including direct antivirals, immunomodulatory and anti-inflammatory agents, anticoagulants, and antiplatelet agents, are being evaluated. However, although some of these have been shown to be effective in treating COVID-19 and reducing mortality, the possibility cannot be excluded at this time that SARS-CoV-2 variants may evade some of these currently effective pharmacological therapies. Their high cost and uneven distribution around the world also make them an unattainable therapeutic option in most low-income countries (23–25).

Here, we have described an exceptional case in which a two-time renal transplant patient with a severe form of COVID-19 was treated with CP. This patient had no additional risk factors beyond her immunocompromised state. First, we monitored the viral load in different biological samples during the CP treatment. In addition, we sequenced the complete genome of the virus, which was assigned to the B.1.1.33 SARS-CoV-2 lineage (**Supplementary Figure 1**). This variant has a higher prevalence in South America, especially Brazil, Argentina, and Uruguay, but is scattered throughout the world (26, 27). The ability to sequence the viral genome in an immunosuppressed patient allows for active monitoring for possible reinfections. It is worth noting that the rate of recurrence of infection in these patients might be higher than in non-immunocompromised patients (28). After CP treatment, we observed a rapid decrease in salivary SARS-CoV-2 viral load, suggesting that the treatment has an effect on viral transmissibility. A decrease was observed in the NS sample, with a smaller downward trend. Both samples were negative at 72 h. On the other hand, the viral load in the TA remained stable. This observation is consistent with previous reports showing greater persistence of the SARS-CoV-2 viral load in lower respiratory tract samples than in upper respiratory tract samples (29, 30). It is important to note that some immunocompromised patients may test negative for SARS-CoV-2 in NS samples, even though a latent viral source may persist for long periods of time. In fact, general persistence of SARS-CoV-2 has been reported in immunocompromised patients (16). The decrease in viral load in this case was consistent with the increase in antibody levels after administration of the CP transfusion doses (**Figure 2A**). The absence of antibodies before CP administration supports the idea that the rapid recovery of the patient was due to the treatment received (**Figure 2B**). We also observed a rapid decrease in some of the pro-inflammatory cytokines involved in the cytokine storm after CP transfusion, namely, IFN-γ, IL-6, IL-8, IL-10, and MCP-1 (**Figure 3**).

In summary, the patient’s prognosis improved significantly after CP treatment, based on her clinical condition, oxygen saturation, SARS-CoV-2 viral load, cytokine profile, and plasma anti-viral antibody levels (**Supplementary Material**).

The use of CP may be associated with viral clearance, particularly in patients who have not initiated an endogenous immune response (i.e., serum antibody negative) and have a high viral load at baseline. In this case, the plasma infusion may have triggered the increase in neutralizing antibodies, but may also be responsible for the significant decrease in the levels of several cytokines that control and reduce CS. These findings are of particular importance for developing countries where access to antiviral drugs, monoclonal antibody therapy, or other treatment options is limited, even more so in the context of a pandemic. These therapies represent a simple and affordable option for COVID-19 and other future emerging infectious viral diseases.

## Data availability statement

All data generated or analyzed during this study are included in this published article. All genomes obtained in this study were uploaded to the EpiCoV database in the GISAID initiative under the accession numbers: EPI_ISL_7,457,652, EPI_ISL_7,457,653, and EPI_ISL_7,457,651.

## Ethics statement

Written informed consent was obtained from the individual for the publication of any potentially identifiable images or data included in this article.

## Author contributions

GM and PM conceived the study and designed the analysis. NI, NN, JT, JH, and DF contributed to patient data collection. AF, CS, GI, and MP-G analyzed next-generation sequencing (NGS) data. NN and JT conceived the medical treatment. FA, AF, RA, and DF carried out the processing of samples. FR, HD, OP, MB, and SB contributed to the immune response analysis. FA, AF, NI, RA, NI, DF, GM, and PM contributed to the discussion of all results obtained in this work. FA, AF, NI, GM, and PM wrote the paper. All authors contributed to the article and approved the submitted version.

## Supplementary material

Supplementary Figure 1 Phylogenetic tree showing reference strains of different Nextstrain clades, automatically obtained using Nextclade v.1.6.0. The sequences obtained in this study, corresponding to B.1.1.33 lineage, are depicted by a lilac star clustered within the 20B clade.

Supplementary Table 1 Laboratory parameters. Clinical indexes and laboratory results before and after convalescent plasma transfusion. MCV, mean corpuscular volume of red blood cells; MCH, mean corpuscular hemoglobin; MCHC, mean corpuscular hemoglobin concentration; RDW, red blood cell distribution width. The underlined date indicates when the convalescent plasma was transfused.
