# Supplementary figures and images for: What have we learned from a case of convalescent plasma treatment in a two-time kidney transplant recipient COVID-19 patient? A case report from the perspective of viral load evolution and immune response
Source: Front Nephrol. 2023 Jun 23;3:1132763. doi: 10.3389/fneph.2023.1132763 (PMC10479756; doi:10.3389/fneph.2023.1132763)

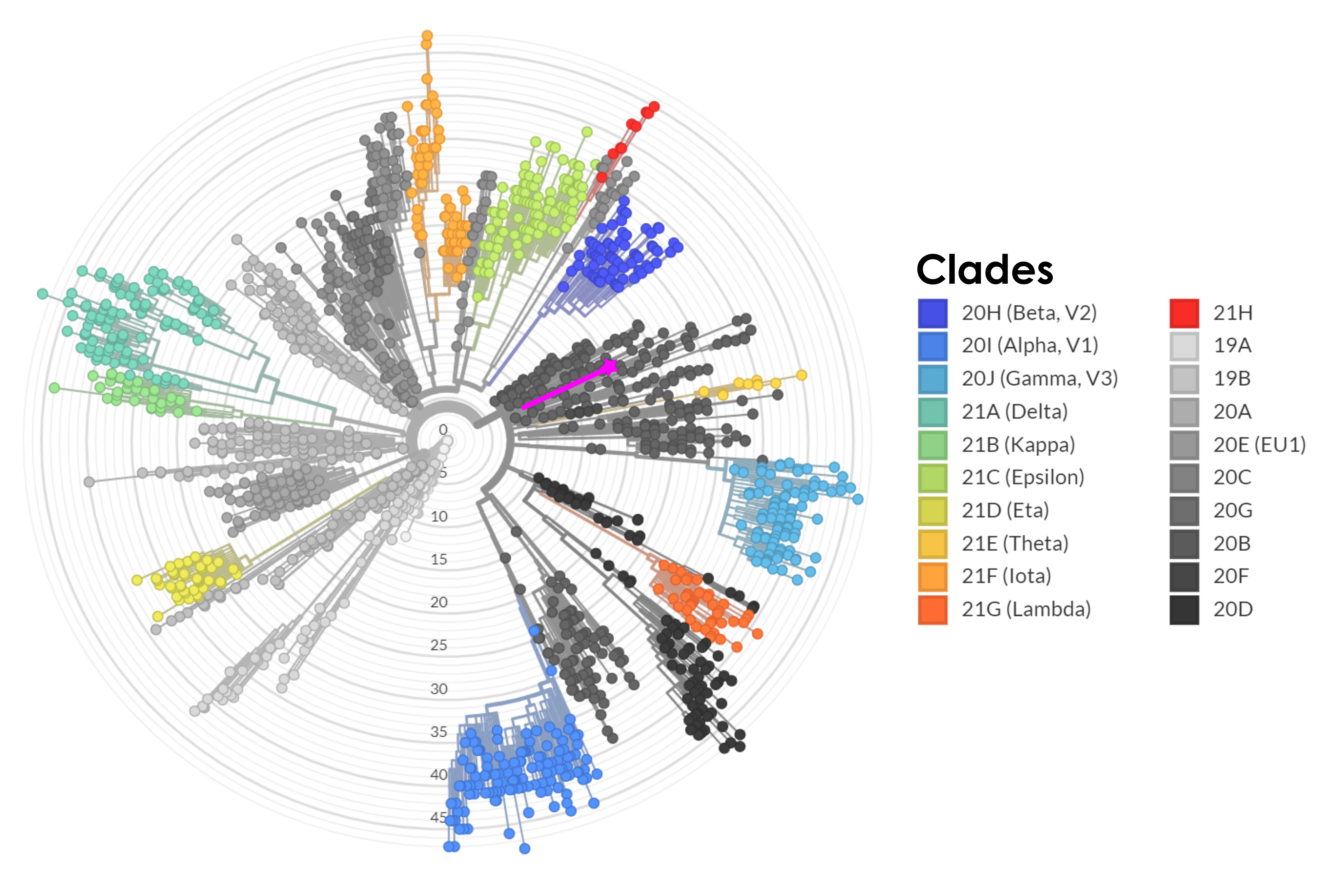

Supplement: Supplementary Figure 1 — Phylogenetic tree showing reference strains of different Nextstrain clades, automatically obtained using Nextclade v.1.6.0. The sequences obtained in this study, corresponding to B.1.1.33 lineage, are depicted by a lilac star clustered within the 20B clade. [file Image_1.jpeg]
